# *ASIP* Promoter Variants Predict the Sesame Coat Color in Shiba Inu Dogs

**DOI:** 10.3390/vetsci9050222

**Published:** 2022-05-03

**Authors:** Stepan N. Belyakin, Daniil A. Maksimov, Maria A. Pobedintseva, Petr P. Laktionov, Dinara Voronova

**Affiliations:** 1 Genomics Laboratory, Institute of Molecular and Cellular Biology, 630090 Novosibirsk, Russia; vift@mcb.nsc.ru (D.A.M.); mapob@mcb.nsc.ru (M.A.P.); laktionov@mcb.nsc.ru (P.P.L.); 2 VetGenomics Laboratory, 630090 Novosibirsk, Russia; 3 Laboratory of Epigenetics, Novosibirsk State University, 630090 Novosibirsk, Russia; 4 Russian Nihonken Hozonkai (NIPPO) Club, 129626 Moscow, Russia; dinara.voronova@gmail.com

**Keywords:** dog coat color genetics, *ASIP* gene, promoter variants, Shiba Inu

## Abstract

Animals exhibit a wide variety of genetically determined coat colors and pigmentation patterns that serve important roles in adaptation and communication. Although the genetics of the main coat colors in dogs have been studied extensively, there are types of coat pigmentation that have not been explained yet. Recently, an association between the variants in the *ASIP* gene Ventral (VP) and Hair Cycle (HCP) promoters with different coat colors in dogs has been established. Here, we used the new findings as a basis to investigate the genetics of the red sesame coat color in Shiba Inu dogs. Our study revealed that red sesame dogs carry a specific heterozygous *ASIP* promoter diplotype, VP2-HCP1/VP2-HCP3, where VP2-HCP1 is responsible for the red coat with a dark overlay, and VP2-HCP3 for a tan point-like pattern. This finding explains the inheritance of this coat color pattern and can be used by breeders to produce dogs with this rare phenotype. A comparison of sesame dogs (VP2-HCP1/VP2-HCP3) to a dog homozygous for the VP2-HCP1 promoter haplotype suggests that the incomplete dominance between the *ASIP* alleles may be involved in the sesame coat formation. These results are in good agreement with the new model explaining how different levels of *ASIP* gene expression affect the regulation of pigment synthesis in melanocytes.

## 1. Introduction

The genetics of coat color in domestic dogs is an example of an extensively characterized biological system with peculiar genetic interactions including several epistases [1,2]. The diversity of the coat colors in dogs results from different combinations of two pigments—dark eumelanin and yellow/red pheomelanin—that are synthesized in melanocytes and loaded into the growing hair [3,4,5].

The Agouti Signaling Protein produced by the *ASIP* gene switches the type of pigment that is produced in melanocytes and thereby plays a central role in coat color formation in different animals [6,7,8]. The ASIP protein is a ligand of the Melanocortin 1 Receptor (MC1R) on the surface of melanocytes. In the absence of ASIP, MC1R promotes eumelanin synthesis. The binding of ASIP to MC1R switches melanocyte to pheomelanin production [9,10,11].

In many animal species, the concentration of ASIP oscillates in the skin on the dorsal side of the body, thereby resulting in a banded pattern of pigmentation along the hair [1,12]. For example, at the starting phase of hair growth, the concentration of ASIP in the skin is low, and eumelanin is predominantly produced in melanocytes and loaded into the hair tip. At some point, the ASIP concentration increases and melanocytes are switched to pheomelanin synthesis, resulting in the deposition of red pigment as the hair continues to grow. When the amount of ASIP reduces again, a new dark band may appear in the hair.

In domestic dogs, the alleles of the *ASIP* gene are known to produce a number of different patterns. When other genes participating in coat color formation are intact, the dominant allele *Ay* leads to a solid red coat pattern. There are variants that produce black-and-tan or saddle tan patterns. The most recessive allele in the set (allele *a*) is responsible for the solid black coat color [13]. The wild-type allele *aw* forms the wolf-sable coat color, characterized by a pheomelanistic ventrum and a banded pattern of hair pigmentation on the dorsum due to ASIP oscillation in the skin.

The different alleles of the *ASIP* gene were previously associated with particular DNA polymorphisms or variants (Table 1). These variants are routinely used in many commercial testing laboratories as markers of the corresponding *ASIP* alleles that explain most of the *ASIP* gene effects on coat pigmentation in dogs. However, according to a recent report [14], these variants are not causative; they are rather linked to the corresponding alleles and, therefore, important exceptions exist that cannot be comprehensively explained by the old tests interrogating the imperfectly associated variants.

Even prior to the discovery of the different *ASIP* promoter variants [14], it was already noted that the widely used commercial tests for the associated variants do not always yield the expected genotypes [19]. The new framework of *ASIP* promoter variants provides a plausible and consistent explanation for the discrepancies observed in the Dreger et al. study [14].

A recent study determined that, apart from the *a* allele, which is indeed the missense loss-of-function mutation (chr24:23393552C>T [20]), other alleles are associated with different promoter haplotypes of the *ASIP* gene [14]. Two *ASIP* gene promoters are known to operate in many animals [12]. The Ventral Promoter (VP) predominantly activates the *ASIP* gene on the ventral part of the body. Its activity gradually decreases toward the dorsal surface. The transcription from VP is permanent and, therefore, the coat color on the ventral surface of the body is normally red due to pheomelanin deposition. Another promoter, which was called Hair Cycle Promoter (HCP), regulates *ASIP* gene activity mostly on the dorsal surface of the body. The activity of this promoter normally circulates, leading to a banded hair pattern [1,12]. It was suggested that these promoters affect *ASIP* gene activity differentially, thereby mediating the phenotypes caused by its different alleles [14].

As has been shown recently, there are at least two different alleles of VP and five alleles of HCP. The combinations of these functional haplotypes accurately determine the different alleles of the *ASIP* gene [14], as summarized in Table 1. Remarkably, the black-and-tan coat pattern in rabbits has been associated with a deletion in the HCP of the *ASIP* gene [21], implying that this deletion causes an effect similar to the *at* allele in dogs.

The hierarchy of the *ASIP* gene alleles can be presented as follows: *Ay* > *Ays* > *aw* > *asa* = *at* > *a*. Two alleles—*asa* and *at*—are semidominant: the dogs with the *asa*/*at* genotype show an intermediate coat pattern between black and tan and saddle tan. The black pigmentation on their heads forms a characteristic “widow’s peak”-shaped pattern [17].

**Figure 1:**
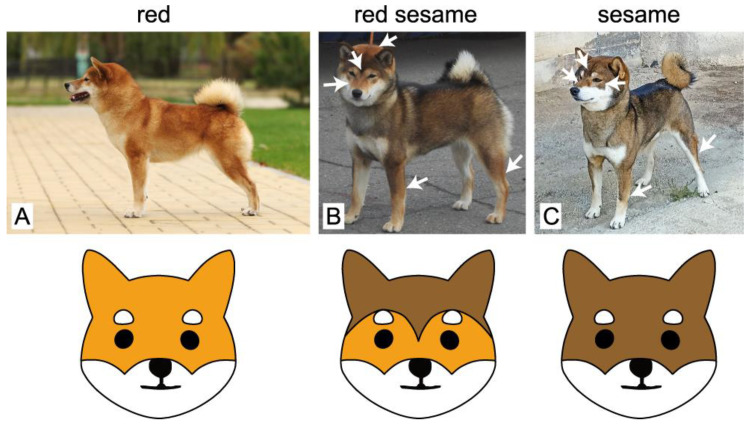
Examples of red (**A**), red sesame (**B**), and sesame (**C**) coat colors in Shiba Inu. Compared to sesame, red sesame dogs have considerably more red pigmentation that is specifically distributed along the body, as indicated with white arrows. A characteristic “widow’s peak”-shaped dark pigmentation between the eyes is shown on the red sesame dog (**B**). Sesame dogs (**C**) typically demonstrate different facial marks (shown with arrows). The illustrations below schematically show the differences in the facial markings of red sesame and sesame dogs.

In Shiba Inu dogs, there is a specific coat pattern that is referred to as sesame (Figure 1). This pattern resembles the black and tan, with the solid black areas replaced by the black overlay. According to the FCI breed standard, there are three distinct subtypes of this coat pattern that are classified as sesame, black sesame, or red sesame. These subtypes are primarily differentiated by the amount of black shading, with red sesame demonstrating considerably more red than sesame or black sesame (Figure 1).

**Figure 2:**
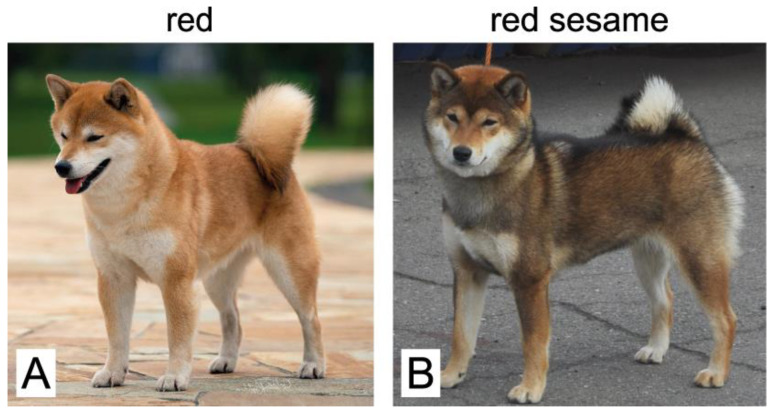
Two dogs that were tested identical *Ay*/*at* with the old tests using the associated markers for *Ay* (chr24:23393351T, chr24:23393514A) and *at* (SINE insertion at chr24:23365297). While the two dogs have identical marker genotypes, they show a striking difference in phenotype: the dog in (**A**) is purely red, while the dog in (**B**) is red sesame.

Most of the dogs in the Shiba Inu breed are red; a low percentage of them are black and tan (also see the statistics in [22]). The sesame coat colors are the rarest ones. The sesame coat pattern in Shiba Inu dogs is an amazing example of the *ASIP* gene’s effects on coat pigmentation. It is known that the sesame coat color is determined by the wild-type allele of the *ASIP* gene, which is very rare in the Shiba Inu breed. In turn, red sesame dogs were shown to possess the double mutation (chr24:23393510G>T and chr24:23393514G>A), as well as the SINE insertion at chr24:23365297, as detected by a widely used genetic test for *ASIP* alleles (see “Associated genetic variant” column in Table 1). The problem is that the dogs with this genotype can be red sesame or red with hardly any black shading (Figure 2). The reason for this variation and the inheritance of the red sesame coat color has remained elusive.

The inheritance of the sesame coat color is unclear and has puzzled breeders and dog owners for decades. Here, we tested whether the promoter-based model of *ASIP* alleles can be applied to explain the sesame coat color formation in Shiba Inu.

## 2. Materials and Methods

### 2.1. Samples

Shiba Inu buccal swabs were used as a source of genomic DNA for this study. The samples were sent to the VetGenomics laboratory by the owners for genetic testing. The dogs used in this study were initially selected using the coat color information indicated in the Shiba Inu pedigree database [23]. Red and sesame dogs were initially selected. The owners of all the dogs in this study signed an agreement for their participation in the study and sent photographs of their dogs to confirm the coat color. Based on this information, the phenotypes of sesame dogs were classified as sesame and red sesame. The resulting cohort included 57 dogs (42 red, 11 red sesame, 3 sesame dogs, and one dog that looked like a red sesame but had a lighter coat). All of these dogs were tested for causative promoter haplotypes [14] and for the associated genetic variants indicated in Table 1.

### 2.2. Genotyping of ASIP Promoters

Genomic DNA was isolated from the swabs and used in PCR (BioMaster HS-Taq PCR-Color (2×) Mastermix, Biolabmix LLC, Novosibirsk, Russian Federation) with the primers for VP and HCP [14]. The genotypes were determined by assessing the lengths of PCR products in 1% agarose gel in 1 × Tris–Acetate–EDTA buffer. The primer pairs and resulting fragment lengths are summarized in Table 2.

### 2.3. Sequencing the MC1R Gene

The coding sequence of the MC1R gene was amplified using the following primers: Forward: 5′-cctcaccaggaacatagcac-3′, Reverse: 5′-ctgagcaagacacctgagag-3′. The 1007 bp PCR product was purified by Polyethylene Glycol (PEG) precipitation [24] in order to remove primers. The purified PCR product was used for Sanger sequencing on a 3500 Genetic Analyser (Applied Biosystems, Foster City, CA, USA).

## 3. Results

We genotyped *ASIP* promoters in Shiba Inu dogs with red and sesame coat colors using a PCR-based approach to detect the different VP and HCP variants (Materials and Methods).

This analysis provided a clear concordance between the coat colors and genotypes determined using the new findings about *ASIP* gene promoters (Table 3). All of the dogs having red coats possessed the most dominant *Ay* allele in their genotypes (in this case, *Ay* = VP1-HCP1). Genotyping of the red sesame dogs revealed the genotype *Ays*/*at* (*Ays* = VP2-HCP1), which clearly distinguished them from the red *Ay*/*at* dogs. The phenotypically darker sesame dogs were confirmed to have the *aw*/*at* genotype. In these cases, the *aw* allele was directly determined by the VP2-HCP2 promoter combination. No dogs with *aw*/*aw* or *Ays/aw* genotypes were found in the present study.

Both VP1-HCP1 (*Ay*) and VP2-HCP1 (*Ays*) combinations are linked to the (chr24:23393510T, chr24:23393514A) variant (Table 1). This explains why routine testing using the previously reported SNV markers could not distinguish between clear red and red sesame dogs, as illustrated in Figure 2.

**Figure 3:**
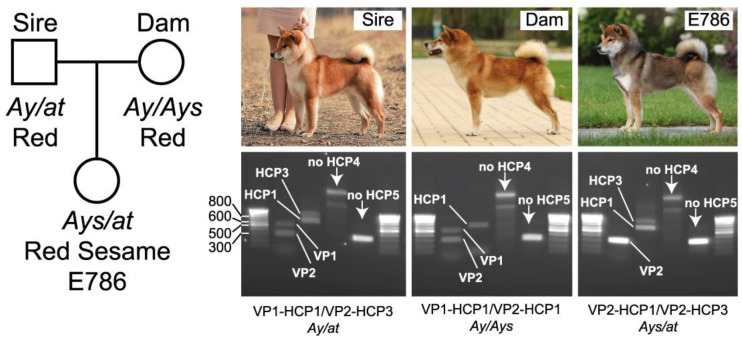
Genotyping of the red sesame dog and her red parents. Red parents and their red sesame daughter were PCR-genotyped for *ASIP* gene promoter haplotypes as described in [14]. The genotyping of the sire revealed the presence of VP1, VP2, HCP1, and HCP3 promoter haplotypes, which uniquely corresponds to the *Ay*/*at* genotype as indicated under the gel photograph [14]. The dam possessed VP1, VP2, and HCP1 promoters, which correspond to the *Ay*/*Ays* genotype. Their daughter inherited *Ays* from dam and *at* from sire, as shown by the presence of VP2, HCP1, and HCP3 variants. The DNA marker sizes are shown in the first photograph.

Our findings explain the puzzling inheritance of the red sesame coat color: red sesame pups may be born if one parent bears the *Ays* allele and another carries the *at* allele. To illustrate this, we genotyped a family that produced a red sesame dog. Both parents were red. The genotyping of *ASIP* gene promoters revealed that the sire had the *Ay*/*at* genotype (VP1-HCP1/VP2-HCP2) while the dam was *Ay*/*Ays* (VP1-HCP1/VP2-HCP1). Their red sesame daughter was confirmed to have the *Ays*/*at* genotype (VP2-HCP1/VP2-HCP3; Figure 3).

To check for the presence of mutations in the *MC1R* gene that could also be involved in the shaded coat color formation, we sequenced this gene in these three dogs. No mutations were detected in the *MC1R* gene, thereby suggesting that mutations in locus E apparently do not have a major impact on the sesame phenotype formation in Shiba Inu.

The typical red sesame Shiba Inu dogs that were used in this study have the *Ays*/*at* genotype. This suggests that an incomplete dominance of the *Ays* allele over the *at* allele could exist. Alternatively, the red sesame coat color would be produced by *Ays* alone and the recessive *at* allele would not be manifested in the phenotype. In the latter scenario, *Ays*/*at* dogs should not be phenotypically different from *Ays*/*Ays* animals.

**Figure 4:**
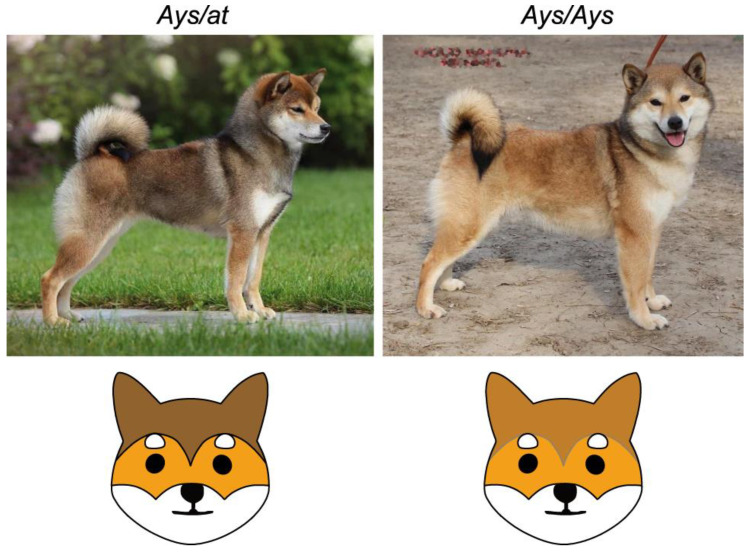
Incomplete dominance of the *Ays* allele over the *at* allele in red sesame Shiba Inu. *Ays*/*at* dog has a typical red sesame coat. The *Ays*/*Ays* dog discovered in this study demonstrates significantly more red in their coat color, although with obvious dark shading, clearly distinguishing it from red Shiba Inu dogs. The illustrations below schematically show the differences in the facial markings of *Ays/at* and *Ays/Ays* dogs.

The *Ays* allele is quite rare in Shiba Inu dogs. However, we identified one dog with the *Ays*/*Ays* genotype (Table 2). As shown in Figure 4, this dog has a dark overlay that is similar to a typical red sesame coat color, but this overlay is generally thinner and much more red pigmentation is observed compared to the red sesame *Ays*/*at* dogs that were analyzed in our study. This example suggests that the *Ays* allele is incompletely dominant over the *at* allele, yet individual variation in allele expressivity cannot be ruled out. A more representative analysis of confirmed *Ays*/*Ays* Shiba Inu dogs would help to validate this.

We also performed the complete sequencing of *MC1R* coding sequences in this *Ays*/*Ays* dog and found two synonymous mutations (chr5:63695055C>T and chr5:63695037C>T). These mutations led to AAG > AAA and TCG > TCA codon substitutions, which did not affect the amino acid sequence of the MC1R protein (Supplementary Material). The frequencies of the resulting codons in the dog genome (AAA—0.437, TCA—0.146) were not dramatically different from the frequencies of the ancestral codons (AAG—0.563, TCG—0.059), thereby suggesting that the translation efficiency would not be significantly affected by these replacements. This analysis indicates that the *MC1R* gene is most probably not involved in the lighter shade of the coat color that is observed in the *Ays*/*Ays* dog.

## 4. Discussion

Here, we used the recently published model of the *ASIP* gene effects on coat pigmentation in dogs [14] in order to explain the inheritance of the red sesame coat color in Shiba Inu dogs.

Our results uncover a peculiar interplay between *ASIP* alleles and the coat color phenotypes. All red sesame Shiba Inu dogs in this study were *Ays/at* heterozygous (VP2-HCP1/VP2-HCP3). Therefore, this coat color appears to be a product of the intricate regulation of the *ASIP* gene in the skin. Two copies of the VP2 promoter ensure the permanent expression of this gene on the ventral side of the body. However, a single copy of the HCP1 promoter apparently is unable to maintain the high level of ASIP protein, which would be sufficient to mask the manifestation of the non-functional HCP3 promoter on the dorsal side of the body.

This suggestion is further supported by the phenotype of the *Ays/Ays* dog, which had a coat color that was similar to but clearly distinct from red sesame. Compared to red sesame dogs, this example could be serve as important evidence of the incomplete dominance of the *Ays* allele over the *at* allele in Shiba Inu. Although this observation is based on a single *Ays/Ays* dog and requires a more representative analysis, it seems to be in line with the previous reports of the interactions between different *ASIP* alleles. Another example of the incomplete dominance of *ASIP* alleles was described in Welsh Corgi Pembroke: *asa*/*at* combination produces a “widow’s peak”-shaped pattern on the dog’s head, which is intermediate between saddle tan and black-and-tan [17]. These results align very well with the new model explaining the action of *ASIP* alleles via the differential activity of Ventral and Hair Cycle promoters [14].

Our work was focused on the genotyping of *ASIP* gene promoters. This design cannot completely exclude the involvement of other genes in sesame coat color. For example, a recently characterized *e^A^* allele of the *MC1R* gene showed a somewhat similar pattern of pigmentation in different dog breeds [25]. As this allele is ancient in canine lineage, it is possible that it also exists in Shiba Inu dogs. However, the direct sequencing of *MC1R* in the red sesame dog detected no mutations within its CDS. Taken together with the perfect correlation between the red sesame coat color and the *Ays*/*at* genotype, this suggests that locus E plays no role in the sesame coat color.

Finally, our conclusions support the recently published promoter-centered model of *ASIP* alleles and provide a useful tool to the cynological community and particularly to Shiba Inu breeders.

## Figures and Tables

**Table 1:** Genetic characteristics of *ASIP* gene alleles.

Allele Name	Causative Promoter Haplotype ^&^	Associated Genetic Variant ^#^	Reference ^§^
**Dominant yellow (allele *Ay*)**	DY: VP1-HCP1	chr24:23393510T and chr24:23393514A	[15]
**Shaded yellow (allele *Ays*) ^£^**	SY: VP2-HCP1	chr24:23393510T and chr24:23393514A	[15]
**Wild type** **(allele *aw*)**	AG: VP2-HCP2	NA	
**Saddle tan** **(allele *asa*)**	BS: VP1-HCP4	SINE insertion at chr24:23365297	[16]
**Black and tan (allele *at*)**	BB: VP2-HCP3-5	SINE insertion at chr24:23365297 and chr24:23252755-70dup	[16] [17]

^&^ Causative promoter haplotypes discovered [14]. Alternative allele abbreviations suggested by [14] state for: DY—Dominant Yellow; SY: Shaded Yellow; AG: Agouti; BS: Black Saddle; BB: Black Back. ^#^ Genomic coordinates according to CanFam3.1 assembly [18]. ^§^ Literature references for the associations indicated in the column “Associated genetic variant”. ^£^ The *Ays* (*Ay, shaded*) name was introduced in this study to distinguish it from the canonical Dominant yellow *Ay* allele. Both of these alleles are associated with the same genetic variant indicated in the column “Associated genetic variant” but they correspond to different causative promoter haplotypes.

**Table 2:** Primers used for VP and HCP variant detection and the resulting fragment lengths.

Primer Pair *	Product Size (bp) in the *ASIP* Promoter Haplotypes						
	VP1	VP2	HCP1	HCP2	HCP3	HCP4	HCP5
5′-AGCATGCTTATGTGGCATGT-3′ 5′-CGCTCTTTCAATGTGATTGG-3′	475	300	-	-	-	-	-
5′-TTAAAAAGTGAAAGTGAAAAGATAACCC-3′ 5′-CAAAATTCTGGGTGGGCTAA-3′	-	-	600	400	780	-	-
5′-TTAAAAAGTGAAAGTGAAAAGATAACCC-3′ 5′-TCAATGGAAATGGCAGAACA-3′	-	-	-	-	-	860	-
5′-GATTGAAAGCCAAAGGGTGA-3′ 5′-AGAGCAGGCCAGGTTTTACA-3′	-	-	300	300	300	300	400

* The primer sequences were taken from [14].

**Table 3:** Genotyping *ASIP* gene promoter haplotypes * in 56 red and sesame Shiba Inu dogs.

Coat Color	*Ay*/*Ay*	*Ay*/*Ays*	*Ay*/*at*	*Ay*/*aw*	*Ays*/*at*	*aw*/*at*
red	26	7	6	3	-	-
red sesame	-	-	-	-	11	-
sesame	-	-	-	-	-	3

* The combinations of promoter haplotypes indicated in Table 1 were used as the determinants of *ASIP* alleles: *Ay*, *Ays*, *aw*, and *at*.

## Data Availability

Publicly available datasets were analyzed in this study. This data can be found here: http://www.shiba-pedigree.ru (accessed on 21 October 2021).

## Supplementary Materials

The following supporting information can be downloaded at: https://www.mdpi.com/article/10.3390/vetsci9050222/s1, Supplementary File contains the sequence of the *MC1R* from the *Ays/Ays* Shiba Inu dog. The detected polymorphisms are indicated in bold.

## References

[1] Kaelin C.B., Barsh G.S. (2013). Genetics of pigmentation in dogs and cats. Annu. Rev. Anim. Biosci..

[2] Schmutz S.M., Berryere T.G. (2007). Genes affecting coat colour and pattern in domestic dogs: A review. Anim. Genet..

[3] Ando H., Niki Y., Ito M., Akiyama K., Matsui M.S., Yarosh D.B., Ichihashi M. (2012). Melanosomes are transferred from melanocytes to keratinocytes through the processes of packaging, release, uptake, and dispersion. J. Investig. Dermatol..

[4] Tobin D.J. (2008). Human hair pigmentation--biological aspects. Int. J. Cosmet. Sci..

[5] Prota G. (1980). Recent advances in the chemistry of melanogenesis in mammals. J. Investig. Dermatol..

[6] Barsh G.S. (2006). Regulation of Pigment Type Switching by Agouti, Melanocortin Signaling, Attractin, and Mahoganoid. The Pigmentary System.

[7] Chandramohan B., Renieri C., La Manna V., La Terza A. (2013). The alpaca agouti gene: Genomic locus, transcripts and causative mutations of eumelanic and pheomelanic coat color. Gene.

[8] Han J.L., Yang M., Yue Y.J., Guo T.T., Liu J.B., Niu C.E., Yang B.H. (2015). Analysis of agouti signaling protein (ASIP) gene polymorphisms and association with coat color in Tibetan sheep (Ovis aries). Genet. Mol. Res..

[9] Millar S.E., Miller M.W., Stevens M.E., Barsh G.S. (1995). Expression and transgenic studies of the mouse agouti gene provide insight into the mechanisms by which mammalian coat color patterns are generated. Development.

[10] Ollmann M.M., Lamoreux M.L., Wilson B.D., Barsh G.S. (1998). Interaction of Agouti protein with the melanocortin 1 receptor in vitro and in vivo. Genes Dev..

[11] Lu D., Willard D., Patel I.R., Kadwell S., Overton L., Kost T., Luther M., Chen W., Woychik R.P., Wilkison W.O. (1994). Agouti protein is an antagonist of the melanocyte-stimulating-hormone receptor. Nature.

[12] Vrieling H., Duhl D.M., Millar S.E., Miller K.A., Barsh G.S. (1994). Differences in dorsal and ventral pigmentation result from regional expression of the mouse agouti gene. Proc. Natl. Acad. Sci. USA.

[13] Willis M.B. (1989). Genetics of the Dog.

[14] Bannasch D.L., Kaelin C.B., Letko A., Loechel R., Hug P., Jagannathan V., Henkel J., Roosje P., Hytonen M.K., Lohi H. (2021). Dog colour patterns explained by modular promoters of ancient canid origin. Nat. Ecol. Evol..

[15] Berryere T.G., Kerns J.A., Barsh G.S., Schmutz S.M. (2005). Association of an Agouti allele with fawn or sable coat color in domestic dogs. Mamm. Genome.

[16] Dreger D.L., Schmutz S.M. (2011). A SINE insertion causes the black-and-tan and saddle tan phenotypes in domestic dogs. J. Hered..

[17] Dreger D.L., Parker H.G., Ostrander E.A., Schmutz S.M. (2013). Identification of a mutation that is associated with the saddle tan and black-and-tan phenotypes in Basset Hounds and Pembroke Welsh Corgis. J. Hered..

[18] Lindblad-Toh K., Wade C.M., Mikkelsen T.S., Karlsson E.K., Jaffe D.B., Kamal M., Clamp M., Chang J.L., Kulbokas E.J., Zody M.C. (2005). Genome sequence, comparative analysis and haplotype structure of the domestic dog. Nature.

[19] Dreger D.L., Anderson H., Donner J., Clark J.A., Dykstra A., Hughes A.M., Ekenstedt K.J. (2020). Atypical Genotypes for Canine Agouti Signaling Protein Suggest Novel Chromosomal Rearrangement. Genes.

[20] Kerns J.A., Newton J., Berryere T.G., Rubin E.M., Cheng J.F., Schmutz S.M., Barsh G.S. (2004). Characterization of the dog Agouti gene and a nonagoutimutation in German Shepherd Dogs. Mamm. Genome.

[21] Letko A., Ammann B., Jagannathan V., Henkel J., Leuthard F., Schelling C., Carneiro M., Drogemuller C., Leeb T. (2020). A deletion spanning the promoter and first exon of the hair cycle-specific ASIP transcript isoform in black and tan rabbits. Anim. Genet..

[22] Dreger D.L., Hooser B.N., Hughes A.M., Ganesan B., Donner J., Anderson H., Holtvoigt L., Ekenstedt K.J. (2019). True Colors: Commercially-acquired morphological genotypes reveal hidden allele variation among dog breeds, informing both trait ancestry and breed potential. PLoS ONE.

[23] Shiba Inu Pedigree DataBase. http://www.shiba-pedigree.ru.

[24] Lis J.T. (1980). Fractionation of DNA fragments by polyethylene glycol induced precipitation. Methods Enzymol..

[25] Anderson H., Honkanen L., Ruotanen P., Mathlin J., Donner J. (2020). Comprehensive genetic testing combined with citizen science reveals a recently characterized ancient MC1R mutation associated with partial recessive red phenotypes in dog. Canine Med. Genet..

