# Supplementary material for: ASIP Promoter Variants Predict the Sesame Coat Color in Shiba Inu Dogs
Source: Vet Sci. 2022 May 3;9(5):222. doi: 10.3390/vetsci9050222 (PMC9146165; doi:10.3390/vetsci9050222)
Supplement: Supplementary file 1 [file vetsci-09-00222-s001.zip › vetsci-1630865-supplementary.pdf]

ATGTCTGGGCAGGGCCCCCAGAGAAGGCTGCTGGGCTCTCTCAATGGCACCTCCCCAGCCACCCCTCACTTCGAGC
TGGCTGCCAACCAGACCGGGCCCCGGTGCCTGGAGGTGTCCATTCCCGACGGGCTGTTCCTCAGCCTGGGGCTGG
TGAGCGTTGTGGAAAATGTGCTGGTGGTGGCCGCCATTGCCAA**[G>A]**AACCGCAACCTGCACTC**[G>A]**CCCATGT
ATTACTTCATCGGTTGCCTGGCTGTGTCCGACCTGCTGGTGAGCGTGAGCAATGTGCTGGAGACGGCCGTCATGCT
GCTGGTGGCGGCAGGCGCCTTGGCTGCTCAGGCTGCTGTGGTGCAGCAGCTGGACGACATCATTGACGTGCTCAT
CTGTGGTTCCATGGTATCCAGCCTCTGCTTCCTGGGCGCCATTGCCGTGGACCGCTACCTCTCCATCTTCTACGCGC
TGCGATACCACAGCATCGTCACACTCCCGCGGGCGTGGCGGGCCATCTCCGCTATCTGGGTGGCTAGCGTCCTCTC
CAGCACGCTCTTCATTGCCTACTACAATCACACGGCCGTCCTGCTTTGTCTTGTCAGCTTCTTTGTAGCCATGCTGGT
GCTCATGGCAGTGCTGTACGTCCACATGCTTGCCCGCGCCTGCCAGCACGCCCGAGGTATTGCCCGGCTCCATAAG
AGGCAGCACTTCATCCCCCAGGGCTTTGGCCTCAAGGGCGCTGCCACACTCACTATCCTGCTGGGCATTTTCTTTCT
CTGCTGGGGCCCCTTCTTCTTGCACCTCTCACTCGTGGTCCTCTGCCCTCAACACCCCATCTGTGGCTGCGTCTTTCA
GAACTTCAACCTCTTCCTCACCCTCATCATCTGCAACTCCATCATTGACCCCTTCATCTACGCCTTCCGCAGCCAGGA
GCTCCGAAAGACTCTCCAAGAGGTAGTGCTATGTTCCTGGTGA
